# Lung adenocarcinoma and squamous cell carcinoma difficult for immunohistochemical diagnosis can be distinguished by lipid profile

**DOI:** 10.1038/s41598-023-37848-w

**Published:** 2023-07-26

**Authors:** Takashi Yamashita, Yusuke Takanashi, Asuka Uebayashi, Mikako Oka, Kiyomichi Mizuno, Akikazu Kawase, Soho Oyama, Takuya Kitamoto, Minako Kondo, Shiho Omori, Hong Tao, Yutaka Takahashi, Takumi Sakamoto, Tomoaki Kahyo, Haruhiko Sugimura, Mitsutoshi Setou, Norihiko Shiiya, Kazuhito Funai

**Affiliations:** 1 First Department of Surgery, Hamamatsu University School of Medicine, 1-20-1 Handayama, Higashi Ward, Hamamatsu, Shizuoka 431-3192 Japan; 2 Department of Cellular and Molecular Anatomy, Hamamatsu University School of Medicine, 1-20-1 Handayama, Higashi Ward, Hamamatsu, Shizuoka 431-3192 Japan; 3 Department of Tumor Pathology, Hamamatsu University School of Medicine, 1-20-1 Handayama, Higashi Ward, Hamamatsu, Shizuoka 431-3192 Japan; 4 Advanced Research Facilities and Services, Hamamatsu University School of Medicine, 1-20-1 Handayama, Higashi Ward, Hamamatsu, Shizuoka 431-3192 Japan; 5 Preppers Co. Ltd., 1-23-17 Kitashinagawa, Shinagawa Ward, Tokyo, 140-0001 Japan; 6 MS-Solutions Co. Ltd., 2-18-13, Ogawanishimachi, Kodaira, Tokyo 187-0035 Japan; 7 International Mass Imaging Center, Hamamatsu University School of Medicine, 1-20-1 Handayama, Higashi Ward, Hamamatsu, Shizuoka 431-3192 Japan; 8 Department of Systems Molecular Anatomy, Institute for Medical Photonics Research, Preeminent Medical Photonics Education and Research Center, 1-20-1 Handayama, Higashi Ward, Hamamatsu, Shizuoka 431-3192 Japan

**Keywords:** Cancer, Lung cancer, Cancer, Surgical oncology, Cancer, Mass spectrometry

## Abstract

In patients with unresectable non-small cell lung cancer, histological diagnosis is frequently based on small biopsy specimens unsuitable for histological diagnosis when they are severely crushed and do not retain their morphology. Therefore, establishing a novel diagnostic method independent of tissue morphology or conventional immunohistochemistry (IHC) markers is required. We analyzed the lipid profiles of resected primary lung adenocarcinoma (ADC) and squamous cell carcinoma (SQCC) specimens using liquid chromatography-tandem mass spectrometry. The specimens of 26 ADC and 18 SQCC cases were evenly assigned to the discovery and validation cohorts. Non-target screening on the discovery cohort identified 96 and 13 lipid peaks abundant in ADC and SQCC, respectively. Among these 109 lipid peaks, six and six lipid peaks in ADC and SQCC showed reproducibility in target screening on the validation cohort. Finally, we selected three and four positive lipid markers for ADC and SQCC, demonstrating high discrimination abilities. In cases difficult to diagnose by IHC staining, [cardiolipin(18:2_18:2_18:2_18:2)-H]^−^ and [triglyceride(18:1_17:1_18:1) + NH4]^+^ showed the excellent diagnostic ability for ADC (sensitivity: 1.00, specificity: 0.89, accuracy: 0.93) and SQCC (sensitivity: 0.89, specificity: 0.83, accuracy: 0.87), respectively. These novel candidate lipid markers may contribute to a more accurate diagnosis and subsequent treatment strategy for unresectable NSCLC.

## Introduction

Lung cancer is the leading cause of cancer-related deaths worldwide. Non-small-cell lung cancers (NSCLCs) account for approximately 85% of all lung cancers. Nearly 70% of patients with advanced-stage NSCLC are subjected to systemic chemotherapies, molecular targeted therapies, or immune checkpoint inhibitors^1^. Adenocarcinoma (ADC) and squamous cell carcinoma (SQCC) are the two most common histological subtypes of NSCLCs. As optimal drug selection in chemotherapies is tailored according to the histological subtypes, the precise distinction between ADC and SQCC is essential^2^.

For advanced-stage lung cancer patients for whom radical surgery cannot be applied, the histological diagnosis should be based on small biopsy specimens obtained by transbronchial biopsy (TBB) or fine-needle aspiration (FNA). Such small biopsy specimens often be unsuitable for histological diagnosis when they are severely crushed and do not retain morphology^3^. In most NSCLCs, immunohistochemistry (IHC) markers, such as thyroid transcription factor-1 (TTF-1) for ADC and p40 for SQCC, reliably distinguish lung ADC and SQCC, even in small specimens^4^. However, when diagnosing tumor tissues that exhibit focal expression of these IHC markers, small biopsy specimens may not accurately reflect TTF-1 and p40 expressions: such tumors are classified as “non-small cell carcinoma not otherwise specified”^5^. Furthermore, distinguishing poorly differentiated ADCs from SQCCs with low IHC marker expression levels is extremely difficult. Thus, the establishment of a novel diagnostic method that is independent of tissue morphology or conventional IHC markers is expected^6^.

Recent diagnostic methods of NSCLC subtypes based on proteomic and lipidomic analysis by mass spectrometry have been developed in several studies alternatives to the conventional histopathological diagnosis^7,8^. Because diagnosis based on proteomics may reflect the heterogeneous expressions of protein markers like TTF-1 and p40, that will converge to the diagnostic ability of conventional IHC markers^5^, we focused on lipidomics for investigating novel diagnostic markers. Alterations in lipid metabolism are one of the hallmarks of cancer tissues^6^, and drastic differences in lipid profiles reflect different NSCLC subtypes^8,9^. Thus, several studies have suggested lipid markers for the histological subtyping of NSCLC. Although these studies have delivered promising measurable results, the diagnostic ability of these methods on NSCLC cases that are difficult to diagnose by conventional IHC markers has yet to be evaluated.

This study aimed to identify lipid markers that are valid for subtyping NSCLC independent of tissue morphology and IHC markers, by comparing lipid profiles of lung ADC and SQCC tissues using liquid chromatography-tandem mass spectrometry (LC–MS/MS).

## Results

### Patient characteristics

The clinicopathological characteristics of the discovery and validation cohorts are presented in Table 1. Although enrolled cases were assigned randomly, the pathological stage, degree of differentiation, and histologic subtype of ADC showed deviation between the two cohorts.

**Table 1:** Clinicopathological characteristics of the discovery and validation cohorts.

Characteristics	Discovery cohort		Validation cohort	
	ADC (n = 13)	SQCC (n = 9)	ADC (n = 13)	SQCC (n = 9)
Median age (range)	70 (48–88)	71 (54–83)	72 (49–89)	73 (59–83)
Sex				
Male	10 (76.9%)	8 (88.9%)	9 (69.2%)	9 (100%)
Female	3 (23.1%)	1 (11.1%)	4 (30.8%)	0
Smoking history				
Never	3 (23.1%)	0	5 (38.5%)	0
Former/current	10 (76.9%)	9 (100%)	8 (61.5%)	9 (100%)
Pathological stage				
I A	4 (30.8%)	4 (44.4%)	9 (69.2%)	5 (55.6%)
I B	4 (30.8%)	1 (11.1%)	2 (15.4%)	4 (44.4%)
IIA	0	1 (11.1%)	0	0
IIB	4 (30.8%)	3 (33.3%)	1 (7.7%)	0
IIIA	1 (7.7%)	0	1 (7.7%)	0
Degree of differentiation				
Well	2 (15.4%)	2 (22.2%)	4 (30.8%)	2 (22.2%)
Moderate	7 (53.8%)	5 (55.6%)	7 (53.8%)	6 (66.7%)
Poor	4 (30.8%)	2 (22.2%)	2 (15.4%)	1 (11.1%)
Histologic subtype of ADC				
Lepidic	0	–	4 (30.8%)	–
Papillary	8 (61.5%)	–	6 (46.2%)	–
Acinar	3 (23.1%)	–	2 (15.4%)	–
Solid	2 (15.4%)	–	1 (7.7%)	–
Immunohistochemistry				
TTF-1				
Diffuse positive	11 (84.6%)	0	8 (61.5%)	0
Focal positive	1 (7.7%)	1 (11.1%)	3 (23.1%)	2 (22.2%)
Negative	1 (7.7%)	8 (88.9%)	2 (15.4%)	7 (77.8%)
p40				
Diffuse positive	0	6 (66.6%)	0	4 (44.4%)
Focal positive	0	2 (22.2%)	2 (15.4%)	5 (55.6%)
Negative	13 (100%)	1 (11.1%)	11 (84.6%)	0

*ADC* adenocarcinoma, *SQCC* squamous cell carcinoma, *TTF-1* thyroid transcription factor-1.

### LC–MS/MS of the extracted lipids

LC–MS/MS analysis of the lipids extracted from the frozen tissue samples identified 2453 lipid peaks (Supplemental information 2, Sheets 1 and 2). The phosphatidylcholine (PC) (12:0_12:0) level of the samples was significantly correlated with sample weight in the discovery (Spearman’s rank correlation coefficient [rS] = 0.963, *P* < 0.001) and validation (rS = 0.858, *P* < 0.001) cohorts, confirming the high precision of the normalization procedure (Supplemental information 1, Supplemental Fig. 1).

**Figure 1:**
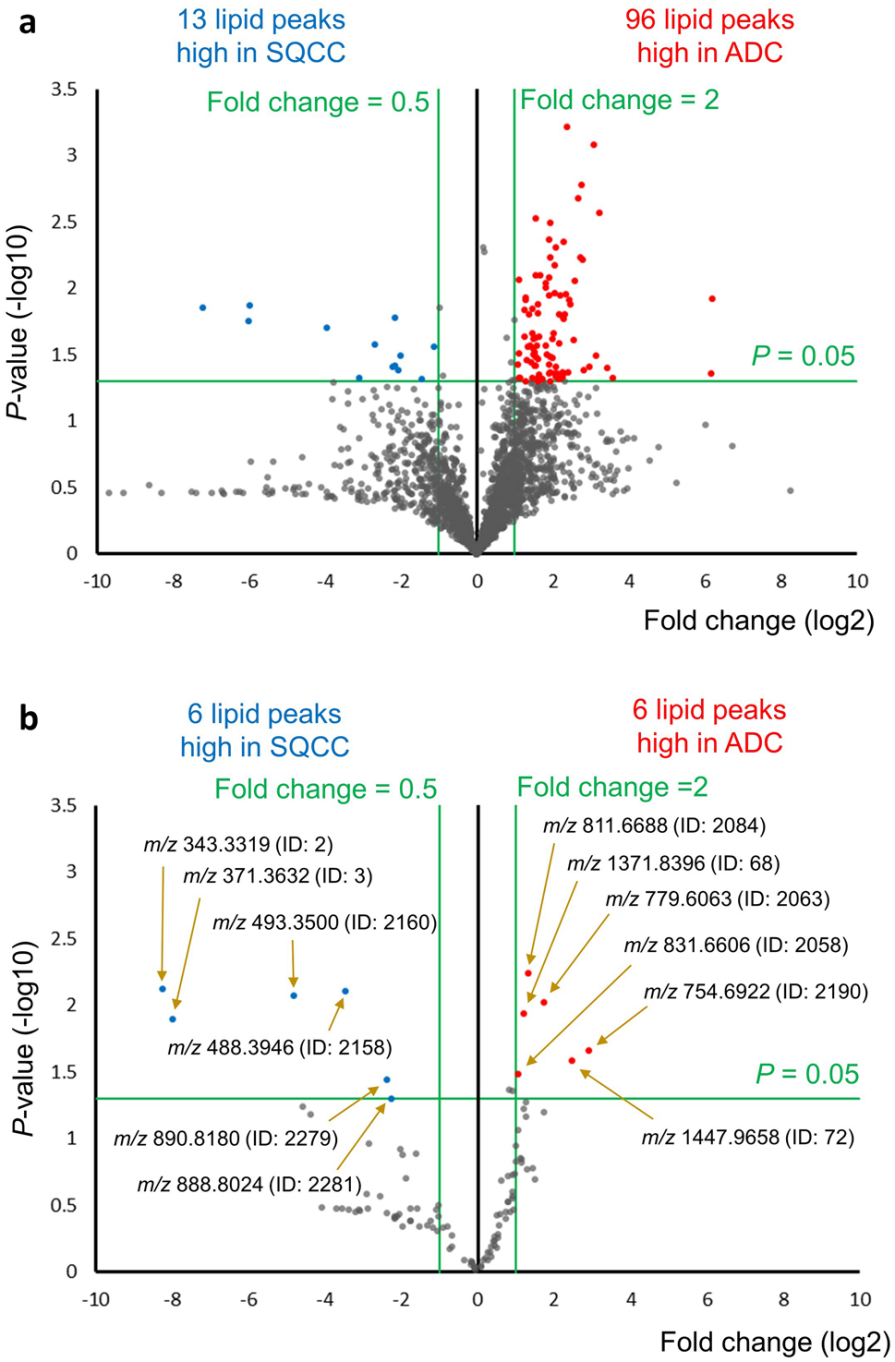
Volcano plots comparing lipidomes of adenocarcinoma (ADC) and squamous cell carcinoma (SQCC) of discovery (**a**) and validation (**b**) cohorts. Based on 2453 lipid peaks identified by liquid chromatography-tandem mass spectrometry analysis, a non-target screening on the discovery cohort identified 96 and 13 lipid peaks high in ADC (red symbols, folding change (FC) ≥ 2.0 [right side of 1 on the horizontal axis], *P*-value < 0.05 [1.301 on the vertical axis]) and SQCC (blue symbols, FC ≤ 0.5 [left side of − 1 on the horizontal axis], *P*-value < 0.05 [1.301 on the vertical axis]), respectively. A subsequent target screening on the validation cohort focusing on these 109 lipid peaks confirmed six and six lipids were reproducibly high in ADC and SQCC, respectively. ADC, Adenocarcinoma; ID, Identity number; *m/z*, Mass-to-charge ratio; SQCC, Squamous cell carcinoma.

### Screening and identification of lipid markers for ADC and SQCC

Based on the 2453 identified lipid peaks, we screened candidate lipid markers for ADC and SQCC using volcano plot analysis. First, a non-target screening in the discovery cohort identified 96 and 13 lipid peaks high in ADC and SQCC, respectively, satisfying the dominance criteria (Fig. 1a). Next, we performed target screening on the validation cohort to examine the reproducibility of the 109 lipid peaks for the dominance criteria. Then, six and six lipid peaks were significantly high in ADC and SQCC, respectively, demonstrating reproducibility for the dominance criteria (Fig. 1b). These lipid peaks are presented with theoretical *m/z* and identity numbers (ID) in Supplemental information 2, Sheets 1 and 2.

We calculated the cut-off values and the area under the receiver operating characteristic (ROC) curves (AUCs) for the 12 lipids to evaluate their discrimination ability for ADC and SQCC. Also, we examined whether these AUCs differed significantly between the discovery and validation cohorts. Among these 12 lipid species, those with a lower limit of the 95% confidence interval for the AUC below 0.5 were considered to have no significant diagnostic ability and were excluded. Thus, among the six lipids abundant in ADC, one lipid peak (ID: 2190) was excluded. Similarly, among the six lipids abundant in SQCC, two lipid peaks (ID: 2160 and 2158) were excluded (Table 2). No significant differences were observed between the AUCs reflecting the reproducibility between the two cohorts.

**Table 2:** AUC rank of candidate lipid markers.

Rank	ID number	Average observed mass	Theoretical mass	Average mass error (ppm)	Ion formula	AUC (95% CI)		*P *value
						Discovery cohort	Validation cohort	
Lipid peaks high in ADC								
** 1**	**2084**	**811.6688**	**811.6688**	**0.0144**	**C47 H92 O6 N2 P1**^**+**^	**0.855 (0.662–1.000)**	**0.821 (0.638–1.000)**	**0.823**
** 2**	**72**	**1447.9658**	**1447.9650**	**0.5933**	**C81 H141 O17 P2**^**-**^	**0.795 (0.594–0.996)**	**0.838 (0.640–1.000)**	**0.723**
** 3**	**68**	**1371.8396**	**1371.8398**	**0.1033**	**C76 H125 O17 P2**^**-**^	**0.786 (0.584–0.989)**	**0.816 (0.618–1.000)**	**0.654**
** 4**	**2058**	**831.6606**	**831.6597**	**1.8091**	**C46 H92 O8 N2 P1**^**+**^	**0.769 (0.561–0.977)**	**0.778 (0.564–0.992)**	**0.945**
** 5**	**2063**	**779.6063**	**779.6062**	**0.2480**	**C45 H84 O6 N2 P1**^**+**^	**0.735 (0.517–0.953)**	**0.855 (0.694–1.000)**	**0.349**
6*	2190	754.6922	754.6919	0.3347	C46 H92 O6 N1^+^	0.692 (0.452–0.932)	0.709 (0.470–0.949)	0.877
Lipid peaks high in SQCC								
** 1**	**3**	**371.3632**	**371.3632**	**0.0588**	**C22 H47 O2 N2**^**+**^	**0.889 (0.754–1.000)**	**0.885 (0.715–1.000)**	**0.810**
** 2**	**2**	**343.3319**	**343.3319**	**0.0560**	**C20 H43 O2 N2**^**+**^	**0.812 (0.609–1.000)**	**0.983 (0.943–1.000)**	**0.111**
** 3**	**2279**	**890.8180**	**890.8171**	**0.9710**	**C56 H108 O6 N1**^**+**^	**0.786 (0.549–1.000)**	**0.838 (0.659–1.000)**	**0.773**
** 4**	**2281**	**888.8024**	**888.8015**	**1.1499**	**C56 H106 O6 N1**^**+**^	**0.778 (0.555–1.000)**	**0.932 (0.821–1.000)**	**0.211**
5*	2160	493.3500	493.3524	4.8553	C29 H49 O6^+^	0.624 (0.319–0.929)	0.709 (0.418–1.000)	0.423
6*	2158	488.3946	488.3946	0.0104	C27 H54 O6 N1^+^	0.590 (0.267–0.913)	0.709 (0.414–1.000)	0.298

*Lipids with 95% CI of AUC that across 0.5 were excluded. *ADC* adenocarcinoma; *AUC* area under the ROC curve; *CI* confidential interval; *ID* identity number; *SQCC* squamous cell carcinoma.

Significant values are in bold.

Thus, MS/MS analyses were conducted on the remaining five and four lipid peaks high in ADC and SQCC, respectively, to identify lipid species. Among the lipid peaks abundant in ADC, three lipid species were confirmed as positive markers for ADC (Supplemental information 1, Supplemental Figs. 2–4): [sphingomyelin (SM)(d20:3_22:0) + H]^+^ (ID: 2084, *m/z*: 811.6688), [cardiolipin (CL)(18:2_18:2_18:2_18:2)-H]^-^ (ID: 72, *m/z*: 1447.9658), and [SM(d40:1) + HCOO]^-^ (ID: 2058, m/z: 831.6597). Two molecules were excluded for the following reasons: *m/z* 1371.8396 (ID: 68) was identified as a dimer of PC(12:0_12:0), while the parental ion peak at *m/z* 779.6063 (ID: 2063) was not observed in the spectral data recorded by the Xcalibur v3.0 Software. Among the lipid peaks abundant in SQCC, four lipid species were confirmed as positive markers for SQCC (Supplemental information 1, Supplemental Figs. 5–7): [anandamide (AEA)(18:1) + NH4] + (ID: 2, m/z: 343.3319), [AEA(20:1) + NH4]^+^ (ID: 3, *m/z*: 371.3632), [triglyceride (TG)(18:1_17:1_18:1) + NH4] + (ID: 2281, m/z: 888.8024) and [TG(17:0_18:1_18:1) + NH4]^+^ (ID: 2279, *m/z*: 890.8180).

**Figure 2:**
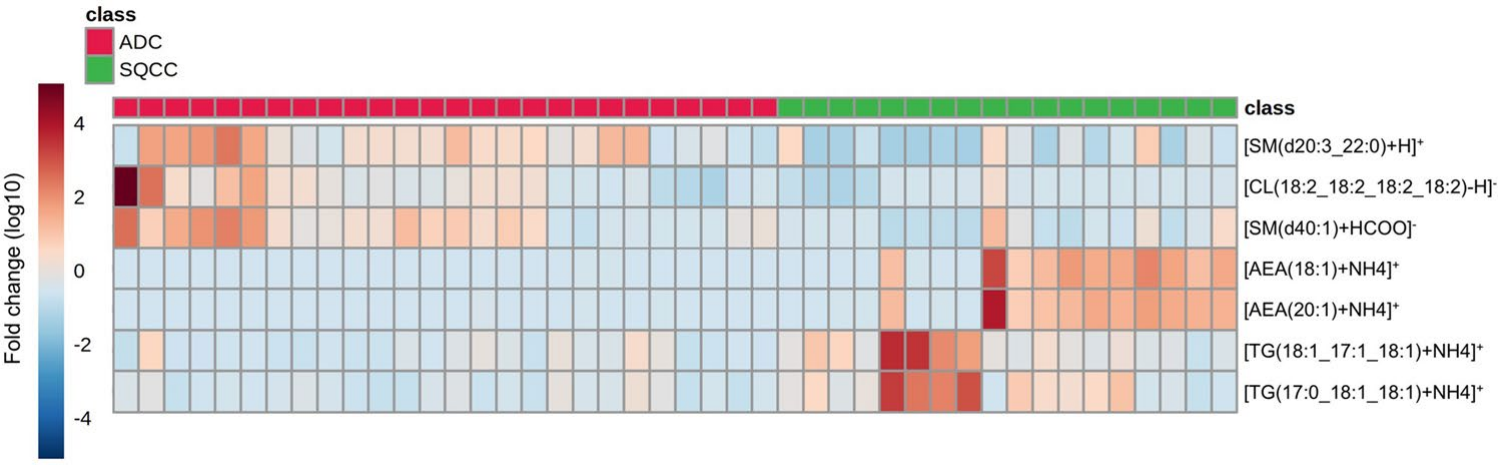
The heatmap shows expression levels of the positive lipid markers for adenocarcinoma (ADC) and squamous cell carcinoma (SQCC). The positive lipid markers for ADC and SQCC were high in most corresponding histological types and low in the other, supporting their validity in discriminating between the two histological types. ADC, Adenocarcinoma; AEA, Anandamide; CL, Cardiolipin; SM, Sphingomyelin; SQCC, Squamous cell carcinoma; TG, triglyceride.

We then re-calculated the cut-off values and AUCs for the three and four positive markers for ADC and SQCC of an entire cohort, which was doubled by combining the discovery and validation cohorts to calculate AUCs close to that of the parent population. The majority of lipid markers, except for [SM(d40:1) + HCOO]^-^ (AUC: 0.784), demonstrated good discrimination abilities with AUC > 0.8 (Table 3). The expression levels of the three lipid markers positive for ADC were high in most ADC cases and low in all SQCC cases. Among the 18 SQCC cases, [AEA(20:1) + NH4]^+^ and [AEA(18:1) + NH4]^+^ were markedly high in 11 cases, whereas [TG(17:0_18:1_18:1) + NH4]^+^ and [TG(18:1_17:1_18:1) + NH4]^+^ were markedly high in four cases (Fig. 2).

**Table 3:** AUC rank of the final candidate lipid markers of the entire cohort.

Rank	ID number	Lipid species	AUC (95% CI)
Positive markers for ADC			
1	2084	[SM(d20:3_22:0) + H]^+^	0.833 (0.703–0.963)
2	72	[CL(18:2_18:2_18:2_18:2)-H]^-^	0.816 (0.685–0.948)
3	2058	[SM(d40:1) + HCOO]^-^	0.784 (0.642–0.926)
Positive markers for SQCC			
1	2	[AEA(18:1) + NH4]^+^	0.895 (0.788–1.000)
2	3	[AEA(20:1) + NH4]^+^	0.894 (0.792–0.996)
3	2281	[TG(18:1_17:1_18:1) + NH4]^+^	0.838 (0.709–0.967)
4	2279	[TG(17:0_18:1_18:1) + NH4]^+^	0.812 (0.668–0.956)

*ADC* adenocarcinoma, *AEA* anandamide, *AUC* area under the *ROC* curve, *CI* confidential interval, *CL* cardiolipin, *ID* identity number, *SM* sphingomyelin, *SQCC* squamous cell carcinoma, *TG* triglyceride.

The correlation between lipids and IHC markers was evaluated (Supplemental information 1, Supplemental Table 1). For ADC, positive lipid markers showed moderate positive and negative correlations with TTF-1 and p40, respectively. In contrast, positive lipid markers for SQCC demonstrated moderate positive and negative correlations with p40 and TTF-1, respectively. The full list of cases is presented with IHC findings and area values of lipid markers in Supplemental information 2, Sheet 3.

### Evaluation of diagnostic abilities of lipid markers in cases difficult to diagnose by IHC

We selected six ADC and nine SQCC cases that were difficult to diagnose by IHC, if we assume diagnosis on small specimens obtained by TBB or FNA. The histopathological characteristics of the patients are presented in Supplemental Table 2. TTF-1 diffuse positivity was not included in the ADC cases, while TTF-1 focal positivity and negativity were present in three cases (50%). Among the SQCC cases, p40 was diffusely positive in only one case (11.1%), while focal positivity and negativity were observed in seven (77.8%) and in one case (11.1%). TTF-1 was focally positive in three cases (33.3%).

**Figure 3:**
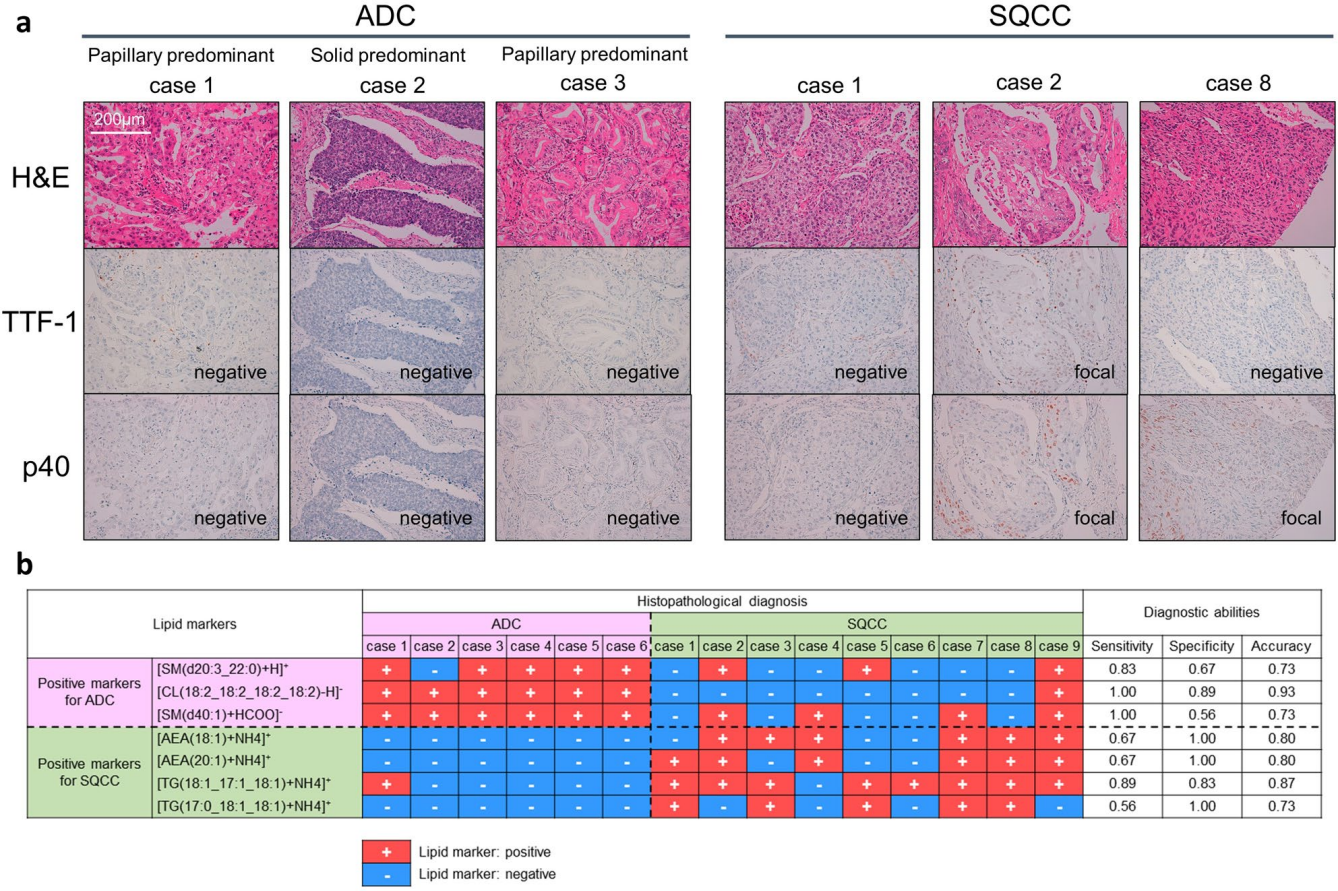
(**a**) Representative histopathological images of adenocarcinoma (ADC) and squamous cell carcinoma (SQCC) cases that were difficult to diagnose by immunohistochemistry (IHC). Thyroid transcription factor-1 was negative in ADC cases, and p40 was negative or focal positive in SQCC cases. Although they were morphologically diagnosed by hematoxylin and eosin stain images obtained from whole surgical specimens, the histological diagnosis would be highly challenging if only small samples with impaired morphological features were available. (**b**) Evaluation of the diagnostic abilities of lipid markers on six ADC and nine SQCC cases that are difficult to diagnose by IHC. Among the seven lipid markers, [cardiolipin(18:2_18:2_18:2_18:2)-H]^-^ demonstrated the highest diagnostic accuracy. ADC, Adenocarcinoma; AEA, Anandamide; CL, cardiolipin; H&E, Hematoxylin and eosin stain; SM, Sphingomyelin; SQCC, Squamous cell carcinoma; TG, Triglyceride; TTF-1, Thyroid transcription factor-1.

Representative histopathological images of ADC and SQCC cases difficult to diagnose by IHC are presented in Fig. 3a. Although the IHC findings of these cases were not typical, they were morphologically diagnosed based on hematoxylin and eosin (H&E) stain images obtained from whole surgical specimens. If only small specimens with impaired morphological features are available in these cases, the histological diagnosis will be highly challenging. The diagnostic abilities of lipid markers for these challenging cases are shown in Fig. 3b. The three positive lipid markers for ADC were highly sensitive (> 0.8) and varied in specificity (0.56–0.89). In contrast, the four positive lipid markers for SQCC were highly specific (> 0.8) and varied in sensitivity (0.56–0.89). All seven lipid markers demonstrated feasible accuracy (0.73–0.93). Overall, ADCs were easy to diagnose correctly, whereas SQCCs tended to be inferior in terms of correct diagnosis. Five SQCC cases (cases 2, 4–6, and 9) that were difficult to diagnose had more than three incorrect lipid marker diagnoses. On the other hand, the other four SQCC cases (cases 1, 3, 7, and 8) that were feasible for diagnosis had less than two that were incorrect. We compared clinicopathological characteristics between “difficult” and “feasible” SQCC groups (Supplemental Table 3). Although no statistically significant differences were detected, poorly differentiated cases were conspicuous in the “difficult” group. The list of cases difficult to diagnose by IHC is presented with IHC findings and area values of lipid markers in the Supplemental information 2, Sheet 4.

With these final seven lipid markers, we have identified candidate lipid markers that are valid for discriminating between lung ADC and SQCC that are difficult to diagnose by IHC markers.

## Discussion

This study identified novel lipid marker candidates for discriminating between lung ADC and SQCC, independent of tissue morphology. Two SM and one CL species were identified as positive markers for ADC, and two AEA and two TG species were identified as positive markers for SQCC. Furthermore, these lipid markers were demonstrated to be valid for discriminating ADC and SQCC cases, which are difficult to diagnose using conventional IHC markers.

Although the patients enrolled in this study were randomly assigned to the discovery and validation cohorts used for lipid screening, differences were observed in the background of the two cohorts due to the small sample size. Thereby, 109 candidate lipid peaks satisfying the significance criteria in the validation cohort were drastically narrowed down to 12 lipid peaks that showed reproducibility. We consider this process enabled efficient screening of candidate markers with universality that can surmount the background difference between the two cohorts. Thus, all the seven final candidate markers showed excellent AUCs ranging from 0.784 to 0.895.

We further evaluated the diagnostic abilities of these seven candidate markers in ADC and SQCC cases that are difficult to diagnose with conventional IHC markers, TTF-1 and p40, particularly assuming diagnosis on small biopsy specimens. Thereupon, positive lipid markers for ADC demonstrated high sensitivity, whereas those for SQCC demonstrated high specificity. With these seven lipid species, we identified the candidate lipid markers that met the aims of this study. We considered that the AUC, sensitivity, specificity, and accuracy of these seven candidate lipid markers were high enough to enable their application as single diagnostic markers. However, a positive marker for ADC can be used as a negative marker for SQCC and vice versa. The combined use of these multiple markers may enable a more precise diagnosis than relying on individual ROC information. Further validation is necessary to utilize the combined use of these candidate markers by establishing predictive models.

In previous studies, mass spectrometry has been utilized to analyze lipids in lung ADC and SQCC to differentiate between the two histological types^8–11^. In LC–MS/MS analyses, molecular species such as SM, PC, phosphatidylinositol, phosphatidylserine, and TG have been identified as candidate markers for differentiation^8,9^. On the other hand, in analyses using imaging mass spectrometry, mainly PC species have been identified as candidate markers for differentiation^10,11^. Among the candidate markers reported in the past, lipid classes such as SM and TG are the same as those identified in our study, but there were no molecules with identical fatty acid side chains; this may be partly due to differences in the equipment and measurement conditions used for lipid analysis. Furthermore, no previous studies have examined the diagnostic ability of identified lipid markers in cases where differentiation is difficult with IHC. The candidate markers we identified are expected to have excellent diagnostic ability even for cases where differentiation is difficult with IHC. Therefore, our findings are considered more responsive to clinical needs compared to conventional studies.

SQCC cases with poor differentiation were difficult to diagnose using positive lipid markers for SQCC correctly. However, as [CL (18:2_18:2_18:2_18:2)-H]^-^ showed the highest diagnostic accuracy among the seven final candidate lipid markers in SQCC cases, the application of this lipid as a negative marker for SQCC may help to overcome this difficulty. Moreover, specimen weight and Brinkman index were not considered to be factors that affected the diagnosis. This indicates the advantages of these markers for consistent diagnostic ability, independent of the specimen size and smoking status.

The seven candidate lipid markers listed in Supplemental Table 1 showed a significant correlation with IHC marker expression, although their correlation coefficients remained moderate. As candidate lipid markers can help to diagnose cases more accurately than IHC markers, the moderate correlations between both factors are compatible.

The positive markers for lung ADC included CL and SM species, and SQCC included AEA and TG species. These results are supported by previous reports and expression differences in enzymes involved in lipid metabolism of lung ADC and SQCC. The expression of human cardiolipin synthase 1 (CRLS1), which is involved in CL biosynthesis^12^, is significantly decreased in lung SQCC compared to normal lung tissue. In contrast, CRLS1 of lung ADC retains the same expression level as normal lung tissue^13^. An mRNA expression analysis based on Kaplan-Meier (KM) plotter demonstrated that sphingomyelin synthase (SGMS) 1 and SGMS2, ​​which encode SM synthase (SMS) 1 and SMS2 involved in SM synthesis^14^, were significantly reduced in lung SQCC compared with normal lung tissue (FC = 0.89, *P* < 0.001 for SMS1 and FC = 0.38, *P* < 0.001 for SMS2). In contrast, in lung ADC, SGMS1 showed no significant difference (FC = 1.20, *P* = 0.448), and SGMS2 was significantly increased (FC = 1.21, *P* < 0.001) (Supplemental Fig. 8). In addition, the mRNA expression of fatty acid amide hydrolase (FAAH) 1, which is involved in the hydrolysis of AEA^15^, was significantly lower in lung SQCC than in normal tissues (FC = 0.63, *P* < 0.001). In contrast, FAAH in ADC showed no significant difference (FC = 0.9, *P* = 0.238) (Supplemental Fig. 9). Lastly, lung SQCC is reported to contain 2.5 times higher TG levels than lung ADC^16^. Our results are consistent with those of previous studies and mRNA expression analyses^12–16^.

This study had several limitations. First, because our study was conducted using a small sample size, differences in the backgrounds of the discovery and validation cohorts were conspicuous. Therefore, the lipid markers identified in this study remain “candidates”. However, we consider that the screened markers that surmounted the background difference between the two cohorts may have a universal diagnostic ability for lung ADC and SQCC. Further validation studies based on larger cohorts are required. Second, the tissue samples used in this study were not obtained by FNA or TBB, but small samples were cut out from the surgical specimens. Therefore, a validation study based on biopsy samples is required. However, because histological diagnosis confirmed by whole tumor sections was available in our study, we could use the rigid diagnostic information as training data to validate the diagnostic ability of lipid markers. In addition, because the weight of the small specimens used for analysis in our study did not exceed that of specimens obtained from transbronchial cryobiopsy^17^, we consider that the small samples in our study well mimicked the actual biopsy samples. Third, we used only a single internal standard, PC (12:0_12:0), for the normalization of identified lipids. In semi-quantitative lipidomics analysis, the extraction and ionization efficiency differ for each lipid class. Therefore, using multiple internal standards corresponding to each lipid class is desirable for more accurate quantification. However, in this study, the lipid classes of interest were unknown, and we could not provide internal standards for all lipid classes. In previous reports, the most abundant lipid class in lung cancer tissues was PC^9^. Therefore, we used non-human PC (12:0_12:0) as the internal standard. This study identified CL, SM, AEA, and TG as lipid class marker candidates to differentiate between lung ADC and SQCC. We will use internal standards corresponding to these lipid classes in future validation studies. Lastly, LC–MS/MS is not a common examination modality used in clinical practice. However, this modality is becoming increasingly important in clinical diagnosis^18–21^. If the introduction of LC–MS/MS as an optional diagnostic modality in a variety of diseases proceeds in the further, the implementation of our lipid markers may be possible.

## Conclusions

We identified seven positive candidate lipid markers that could discriminate between lung ADC and SQCC based on small specimens. These lipid markers demonstrated high diagnostic ability independent of histological morphology and IHC staining, and may contribute to the development of a more accurate diagnosis and treatment strategy for lung cancer.

## Methods

### Patient cohort and tissue samples

The study was conducted in accordance with the Declaration of Helsinki. Retrospective primary lung ADC and SQCC tissue samples intraoperatively obtained from patients who underwent lung resection. Among the lung resection cases between January 2013 and December 2016 at Hamamatsu University Hospital, the cases with written informed consent for a genetic study (institutional ethical board approved No.G14-260) and secondary use were enrolled. Soon after the intraoperative collection, the raw tissue samples were rapidly frozen in liquid nitrogen and stored at − 80 °C. The remaining samples were used for histopathological diagnosis and pathological staging according to the World Health Organization (WHO) criteria and the 8th edition of the TNM classification for lung and pleural tumors^4^, respectively, by experienced pathologists. ADC and SQCC cases that contained no other histological type of tissue were considered eligible. Patients who had received neoadjuvant chemotherapy or radiotherapy were excluded from the study. Finally, 26 ADC and 18 SQCC patients were enrolled in this study.

We divided the ADC and SQCC cohorts evenly into discovery and validation cohorts, respectively (13 ADC and 9 SQCC cases for the respective cohorts). An assignment into the discovery and validation cohorts was performed randomly using a random number table generated by Excel™ (Microsoft, Redmond, WA, USA) (Supplemental information 1, Supplemental Fig. 10).

### Histopathological specimens

Paraffin-embedded tissue blocks were sectioned at three μm thickness and subjected to H&E and IHC staining (TTF-1 for ADC marker and p40 for SQCC marker). H&E staining was performed as follows: sections were deparaffinized with xylene, rehydrated with graded ethanol, and stained with hematoxylin for 15 min. Subsequently, the sections were rinsed with deionized water and treated with acid ethanol (70% ethanol with 0.5% hydrochloric acid) for de-staining. After rinsing with deionized water, eosin staining was performed for 1.5 min, followed by dehydration. Finally, clearing using three-step xylene was followed by cover glass mounting. IHC of TTF-1 (product code: NCL-L-TTF-1, Leica Biosystems Newcastle Ltd, Lincolnshire, USA) and p40 (product code: 718,171, Nichirei Biosciences Inc., Tokyo, Japan) was performed as follows: after deparaffinization and rehydration as noted above, sections were treated with Epitope Retrieval Solution (pH 9) (product code: RE7119, Leica Biosystems Newcastle Ltd, Lincolnshire, USA) at 96 °C for 40 min. Subsequent steps were conducted using an autostainer (Histostainer 48A; Nichirei Biosciences Inc., Tokyo, Japan). The sections were blocked with 3% hydrogen peroxide for 15 min and washed with phosphate-buffered saline (PBS). Incubation with primary antibodies (dilutions were 1:200 for TTF-1 and 1:2 for p40) was performed for 30 min at 25 °C. Next, a secondary antibody reaction using Histofine Simple Stain MAX-PO (product code: 424,151, Nichirei Biosciences Inc., Tokyo, Japan) was performed at room temperature for 30 min, followed by washing with PBS. Detection was performed with diaminobenzidine for 5 min and counterstaining with Mayer’s hematoxylin was performed. Finally, clearing was performed using three-step xylene with subsequent cover-glass mounting.

### Histopathological diagnosis

The histopathological diagnosis of ADC and SQCC was performed by experienced pathologists according to the WHO criteria^4^. TTF-1 and p40 immunoreactivity was scored semi-quantitatively; diffuse reactivity was defined as positive staining of ≥ 50% tumor cells, focal reactivity as 1–49%, and negative reactivity as 0%. The ADC phenotype was confirmed by TTF-1 positivity and p40 negativity. Conversely, the SQCC phenotype was confirmed by p40 positivity and TTF-1 negativity. Histopathological cases in which staining intensities were not different between the two IHC markers were diagnosed based on the morphology observed with H&E staining. The cases that were difficult to diagnose by IHC when assuming small biopsy tissues were selected as follows: IHC staining was atypical (TTF1 negative or p40 positive for ADC, p40 negative or TTF1 positive for SQCC) or very focal.

### Lipid extraction and LC–MS/MS analysis

Lipid extraction from cancer tissues and LC–MS/MS analysis were performed according to previously reported methods^22,23^. Briefly, tissue weight was measured using a Sartorius analytical lab balance CPA224S (Sartorius AG, Göttingen, Germany), and lipids were extracted using a modified Bligh-Dyer method. During extraction, 1,2-dilauroyl-sn-glycero-3-PC (Avanti Polar Lipids, Alabaster, AL), PC(12:0_12:0), was added in proportion to the weight of the tissue samples as an internal standard. Extracted lipids were dried using miVac Duo LV (Genevac, Ipswich, England), and then dissolved in 20 μL of methanol. The dissolved lipids (2 μL) were diluted with methanol proportional to the weight of the tissue samples. We applied 2 μL of the diluted lipid samples to an Acclaim 120 C18 column (150 mm × 2.1 mm, 3 μm) (Thermo Fisher Scientific, Waltham, MA, USA). Components of mobile phase A were water-acetonitrile-methanol (2:1:1 v/v/v), 5 mM ammonium formate, and 0.1% formic acid, while that of mobile phase B were acetonitrile-isopropanol (1:9 v/v), 5 mM ammonium formate, and 0.1% formic acid. The samples were analyzed using a Q Exactive™ Hybrid Quadrupole-Orbitrap™ Mass Spectrometer (Thermo Fisher Scientific, Waltham, MA, USA) with an UltiMate 3000 (Thermo Fisher Scientific, Waltham, MA, USA). MS instrument conditions were as follows: sheath gas flow rate, 50; auxiliary flow rate, 15; sweep gas flow rate, 0; capillary temperature, 250 °C; S-lens RF level, 50; probe heater temperature, 350 °C; and spray voltage of 3.5 kV in positive mode and 2.5 kV in negative mode. Full-MS mode conditions for quantification were as follows: MS scan range, 220–2000; resolution, 70,000; AGC target, 1 × 106 and maximum injection time was 100 ms. For identification, top 5 data-dependent MS2 method with a resolution of 17,500 was used. The AGC target was 1 × 105, and the maximum injection time was 80 ms. Stepped normalized collision energies of 25.5, 30, and 34.5 for the positive mode and 19.5, 30, and 40.5 for the negative mode were applied. Spectral data were acquired in the m/z range of 220–2000 m/z using an Xcalibur v3.0 Software (Thermo Fisher Scientific, Waltham, MA, USA).

### Lipid identification and semi-quantification

The spectral data obtained from the seven ADC and seven SQCC cases analyzed in this study were subjected to LipidSearch™ software version 4.2.13 (Mitsui Knowledge Industry, Tokyo, Japan), and lipid identification was performed with the same parameter settings as reported previously^22,23^. Parameter settings adopted for identification were as follows: database, HCD; retention time, 0.01 min; search type, product_QEX; precursor tolerance, 5.0 ppm; and product tolerance, 8.0 ppm. Identification quality filters of A, B, and C were adopted. Quantification was performed at m/z tolerance of ± 0.01 with retention time range from − 1.0 min to 2.0 min. Identified lipid ions of these 44 cases were aligned with a retention time (RT) tolerance of 0.8 min. Redundant lipid ions identified with different RTs were independent structural isomers (annotated as “Duplication” in Supplemental information 2, Sheets 1 and 2). The area value of a lipid species calculated using LipidSearch™ software was used for semi-quantification and divided by the area value of the internal standard PC(12:0_12:0) in the corresponding case for normalization. The full lists of identified lipid species with normalized area values are presented in Supplemental information, Sheet 1 and 2.

### Data analysis

Screening of the lipid markers for discriminating between ADC and SQCC was conducted by comparing the lipidomes of the ADC and SQCC cohorts. In the discovery cohort, a volcano plot with -log10(*P*-value) for the vertical axis and log2(fold change) for the horizontal axis was drawn using the normalized area values of the identified lipid species. *P*-values were calculated using Welch’s *t*-test by comparing the normalized area values of the respective lipid species between the ADC and SQCC cohorts. The fold change values were calculated by dividing the average normalized area value of the ADC cohort with that of the SQCC cohort for respective lipid species; to divide real numbers, area values of “0” were substituted with the trivial amount “0.0001”. The significance criteria were defined as follows: lipid peaks with *P*-values of < 0.05 and fold change values of ≥ 2.0 were determined as significantly high in the ADC cohort, while lipid peaks with *P*-values of < 0.05 and fold change values of ≤ 0.5 were determined as significantly high in the SQCC cohort. We focused on the screened lipid peaks that met the significance criteria in the discovery cohort and created a volcano plot on the validation cohort only for these lipid peaks. Lipid peaks that satisfied the same significance criteria in the validation cohort with reproducibility were selected as candidates for differentiation factors. A heat map displaying lipid expression levels in each case was created using MetaboAnalyst 5.0 (https://www.metaboanalyst.ca/)^24^.

To evaluate the correlation between lipid differentiation factor levels of a continuous variable and IHC staining of a categorical variable, IHC staining results were substituted into ordinal scales as follows: negative; “0”, focal positive; “1”, diffuse positive; “3”. In addition, the correlation between lipid differentiation factor levels and histopathological diagnosis of a categorical variable was calculated by substituting the latter into ordinal scales as follows: SQCC as “0” and ADC as “1” for evaluating the lipids abundant in ADC, while ADC as “0” and SQCC as “1” for the lipids conversely abundant in SQCC.

The mRNA expression levels in normal and tumor tissues of patients with lung cancer were analyzed based on KM Plotter (https://kmplot.com/analysis/)^25^.

### Statistics

Welch’s *t*-test was used to create volcano plots. To discriminate between ADC and SQCC with lipids, the optimal cut-off values were determined by receiver operating characteristic (ROC) curve analysis. The AUC was calculated to evaluate the discrimination ability of the lipid differentiation factors. DeLong's test was used to determine the difference in the ROC curves between the discovery and validation cohorts for each lipid species. Spearman’s rank correlation analysis was used to evaluate the correlation between the lipid differentiation factor levels and IHC staining or histopathological diagnosis. Except for Welch’s *t*-test, all statistical analyses were performed using R (R Foundation for Statistical Computing, Vienna, Austria, version 3.6.2). Welch’s *t*-test was carried out by the “TTEST” function of Excel™ (Microsoft, Redmond, WA, USA). For all statistical analyses, *P*-values < 0.05 were determined as significant.

### Ethics statement

This study was approved by the Ethics Committee of the Hamamatsu University School of Medicine, Hamamatsu, Japan (#18–264). Patients who were scheduled for tissue collection provided written informed consent preoperatively.

## Supplementary Information

Supplementary Information 1.

Supplementary Information 2.

## Data Availability

The datasets generated and/or analyzed during the current study are available from the corresponding author on reasonable request.
