# Supplementary material for: Lung adenocarcinoma and squamous cell carcinoma difficult for immunohistochemical diagnosis can be distinguished by lipid profile
Source: Sci Rep. 2023 Jul 26;13:12092. doi: 10.1038/s41598-023-37848-w (PMC10372017; doi:10.1038/s41598-023-37848-w)
Supplement: Supplementary file 1 — Supplementary Information 1. [file 41598_2023_37848_MOESM1_ESM.pdf]

Supplemental Figure 1

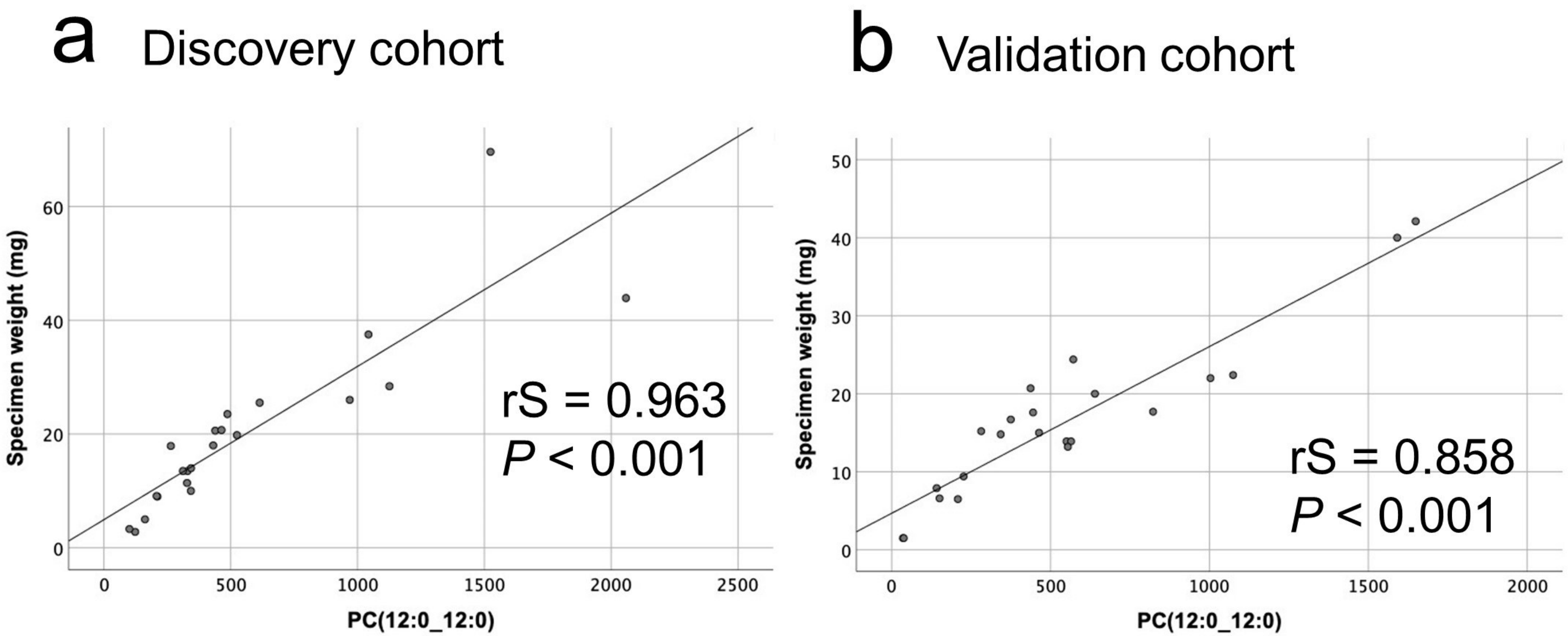

Supplemental Figure 1. PC(12:0_12:0) level of the samples showed significant correlation with sample weight in the discovery (a) and validation (b) cohorts confirming high precision of the normalizing procedure. Abbreviations: PC, phosphatidylcholine; rS, Spearman's rank correlation coefficient.

Supplemental Figure 2

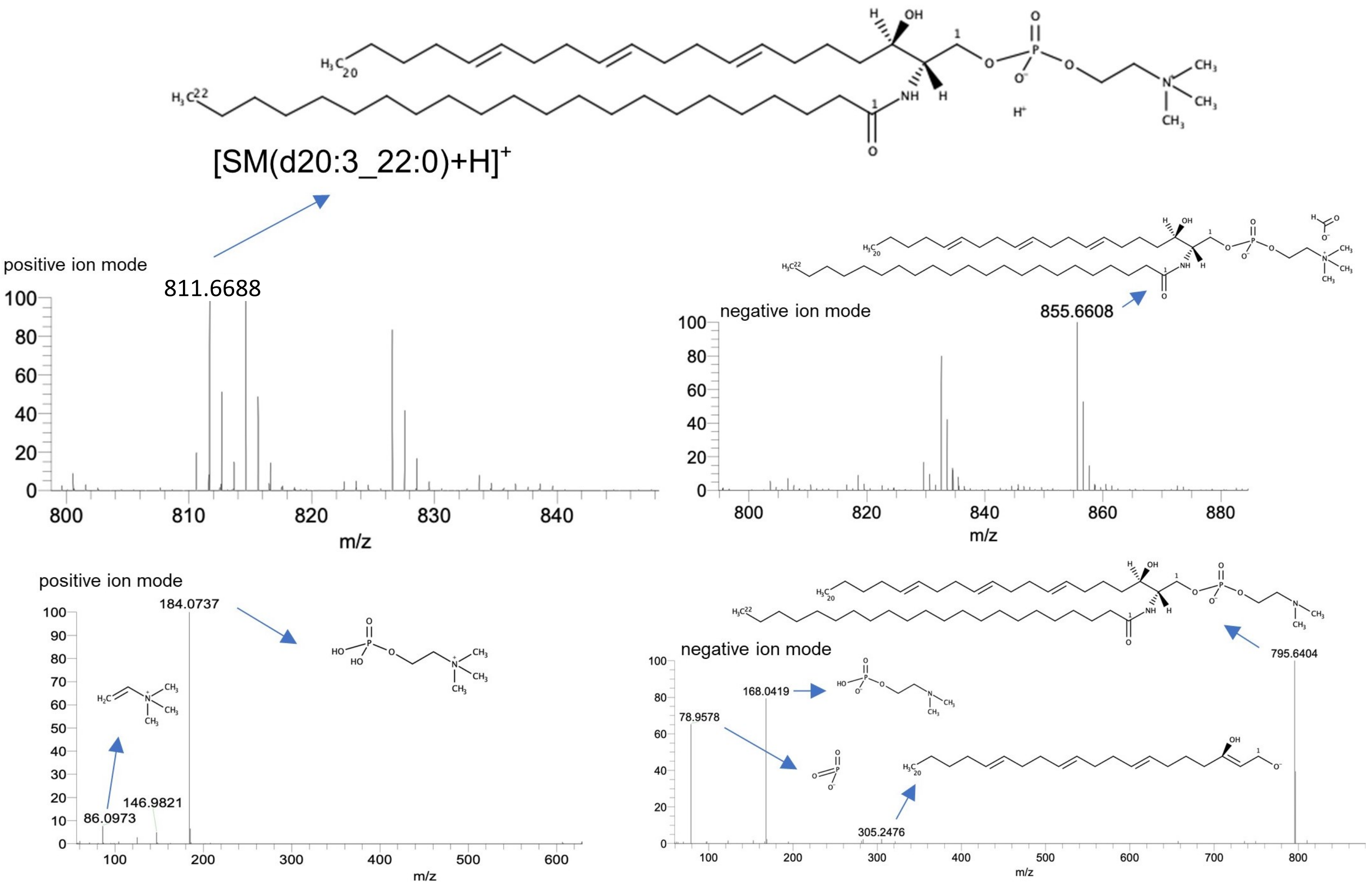

Supplemental Figure 2. MS/MS analysis for *m/z* 811.6688 (ID: 2084). On the positive ion mode, the precursor ion (*m/z* 811.6688) was detected (left upper panel). The fragment ion of *m/z* 184.0737 was a choline head (left lower panel) showing that the precursor ion is a phospholipid. On the negative ion mode, formate adducted precursor ion (*m/z* 855.6608) was detected (right upper panel). The fragment ions contained a sphingosine d20:3 (*m/z* 305.2476) supporting that the precursor ion is a SM (right lower panel). Based on these findings, these fragments were compatible with the product ions from [SM(d20:3_22:0)+H]$^{+}$. Abbreviations: ID, identical number; MS/MS, tandem mass spectrometry; m/z, mass to charge ratio; SM, sphingomyelin.

Supplemental Figure 3

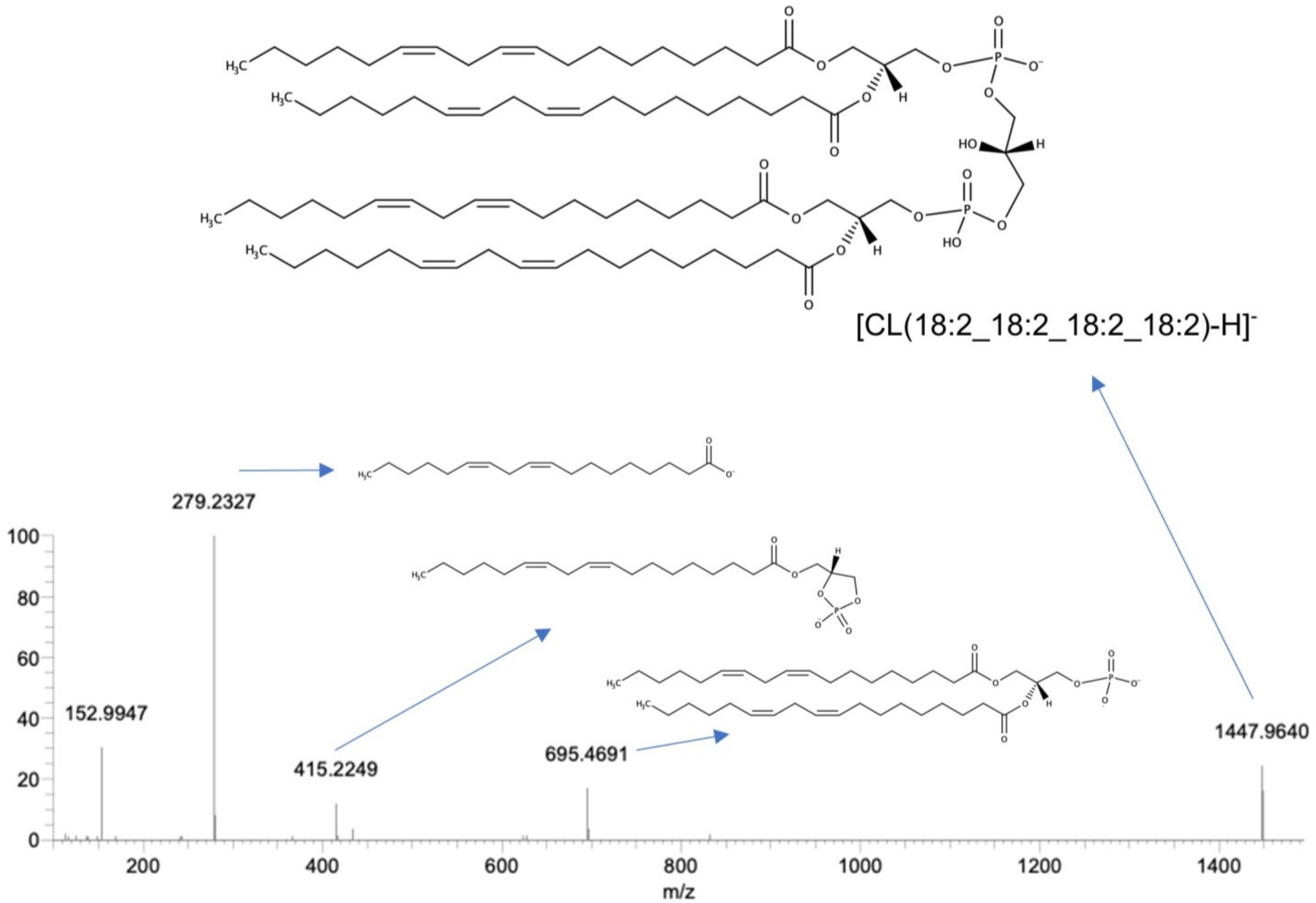

Supplemental Figure 3. MS/MS analysis for *m/z* 1447.9658 (ID: 72). The observed fragment ions were compatible with side chains of [CL(18:2_18:2_18:2_18:2)-H]-.
Abbreviations: CL, cardiolipin; ID, identical number; MS/MS, tandem mass spectrometry; *m/z*, mass to charge ratio.

Supplemental Figure 4

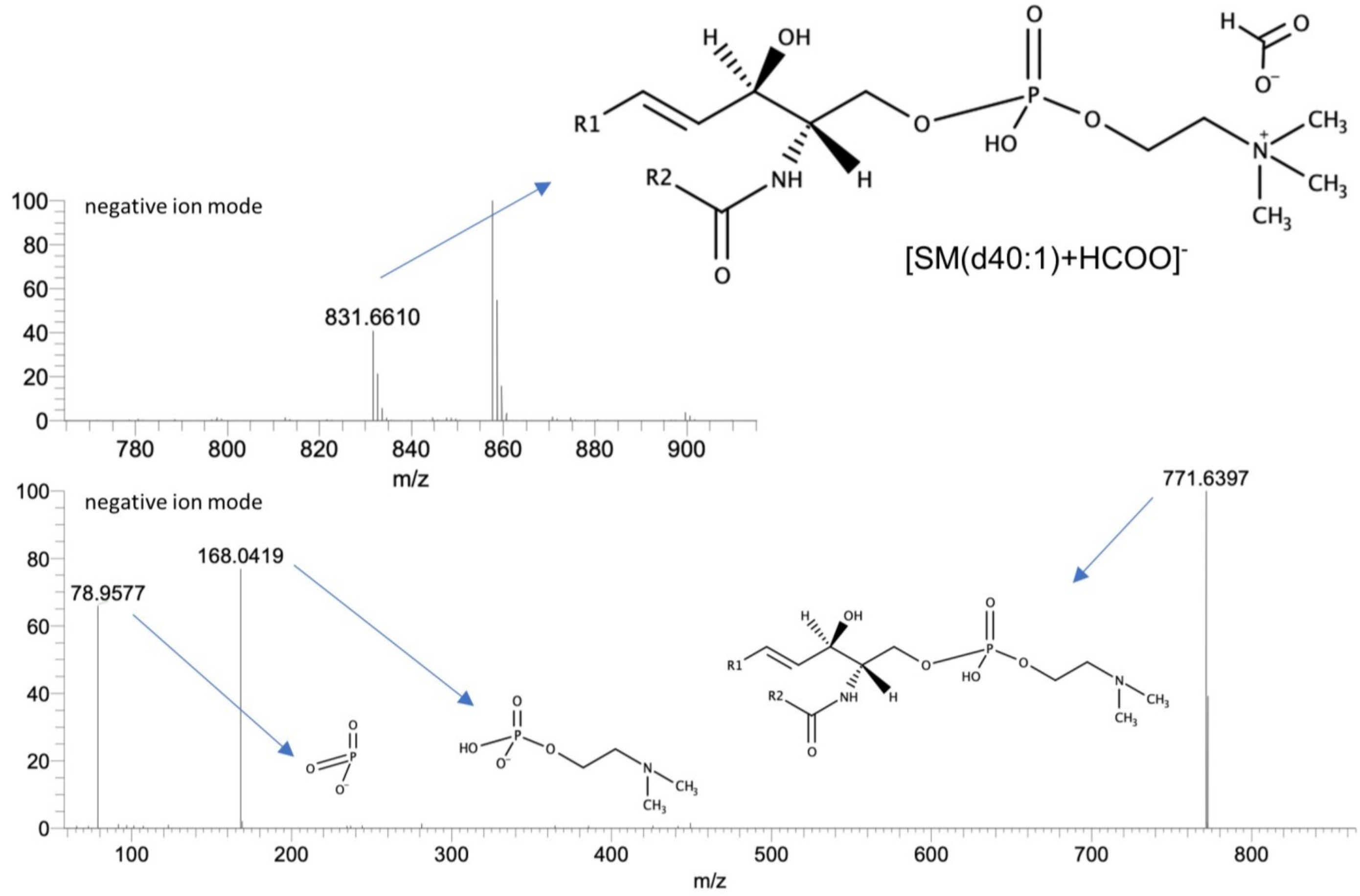

Supplemental Figure 4. MS/MS analysis for *m/z* 831.6597 (ID: 2058). The fragment ions of a choline head (*m/z* 168.0419) and a sphingoid structure (*m/z* 771.6397) were observed (lower panel).
These fragments were compatible with product ions from [SM(d40:1)+HCOO]$^-$.
Its side chains were not identified.
Abbreviations: ID, identical number; MS/MS, tandem mass spectrometry; *m/z*, mass to charge ratio SM, sphingomyelin.

Supplemental Figure 5

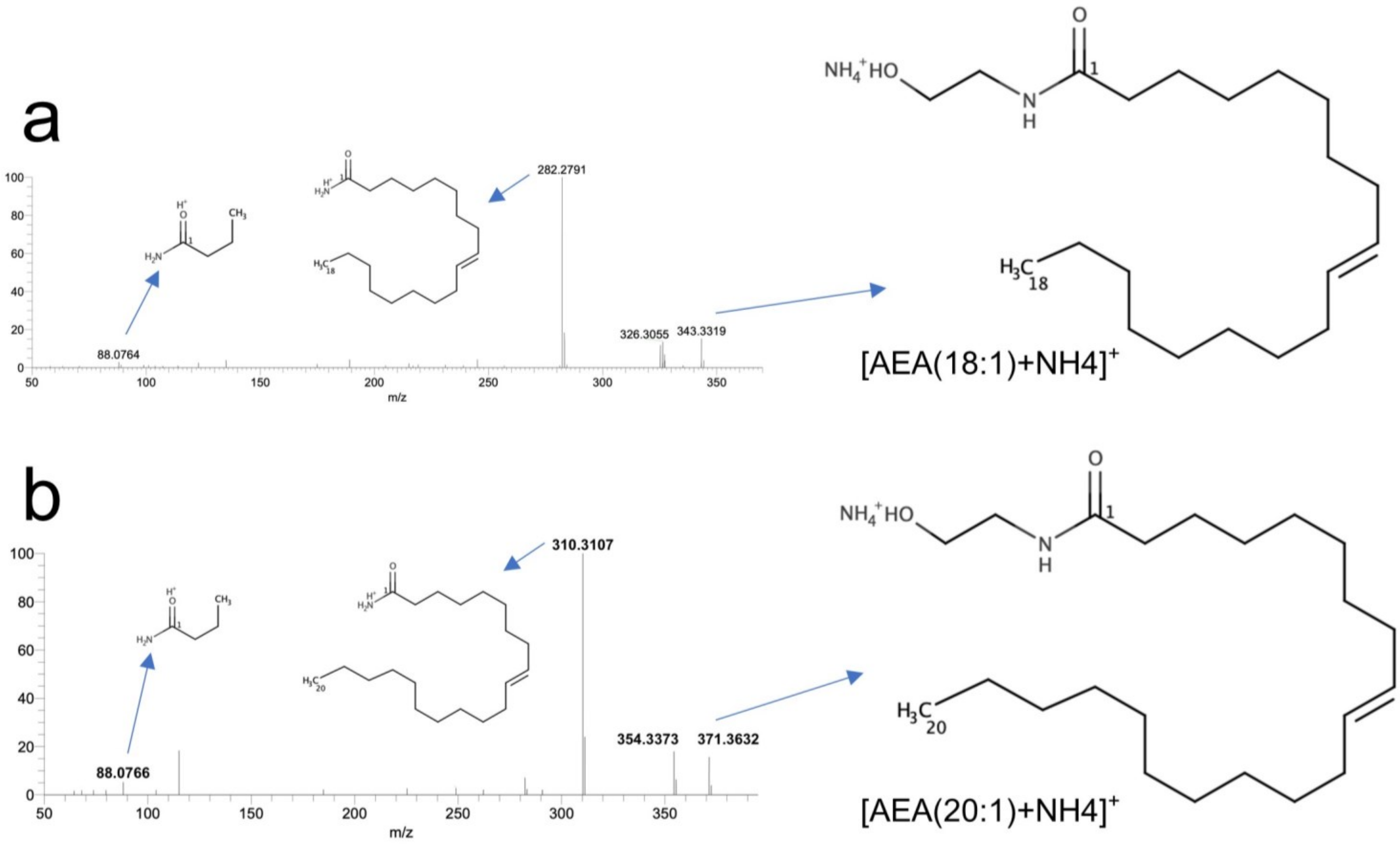

Supplemental Figure 5. MS/MS analysis for *m/z* 343.3319 (ID: 2) and *m/z* 371.3632 (ID: 3). The observed fragment ions were compatible with product ions from [AEA(18:1)+NH4]+ (a) and [AEA(20:1)+NH4]$^{+}$ (b). Abbreviations: AEA, anandamide; ID, identical number; MS/MS, tandem mass spectrometry; *m/z*, mass to charge ratio.

Supplemental Figure 6

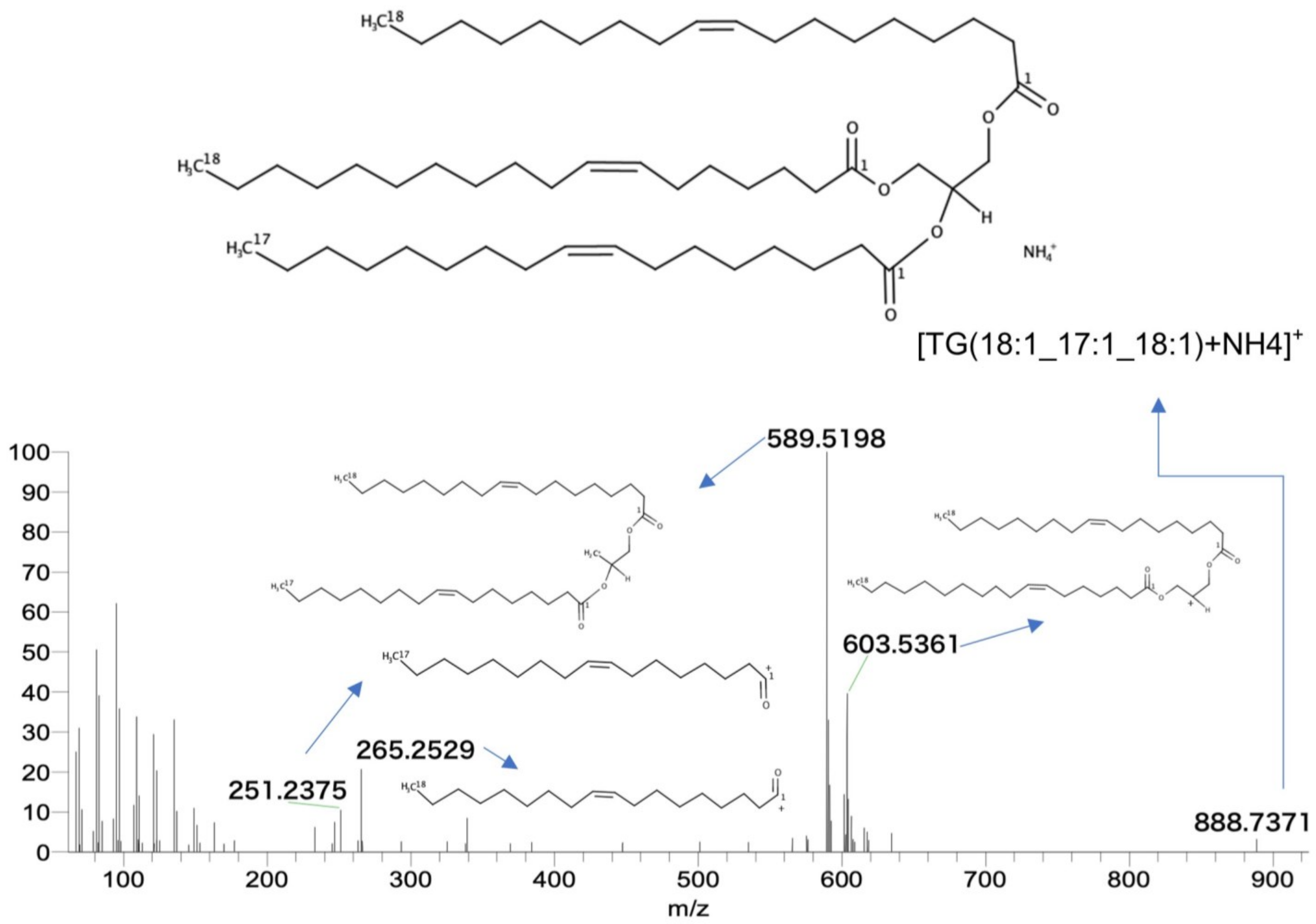

Supplemental Figure 6. MS/MS analysis for *m/z* 888.8024 (ID: 2281). The observed fragment ions were compatible with fatty acid side chains from [TG(18:1_17:1_18:1)+NH4]+.
Abbreviations: ID, identical number; MS/MS, tandem mass spectrometry; *m/z*, mass to charge ratio; TG, triglyceride.

Supplemental Figure 7

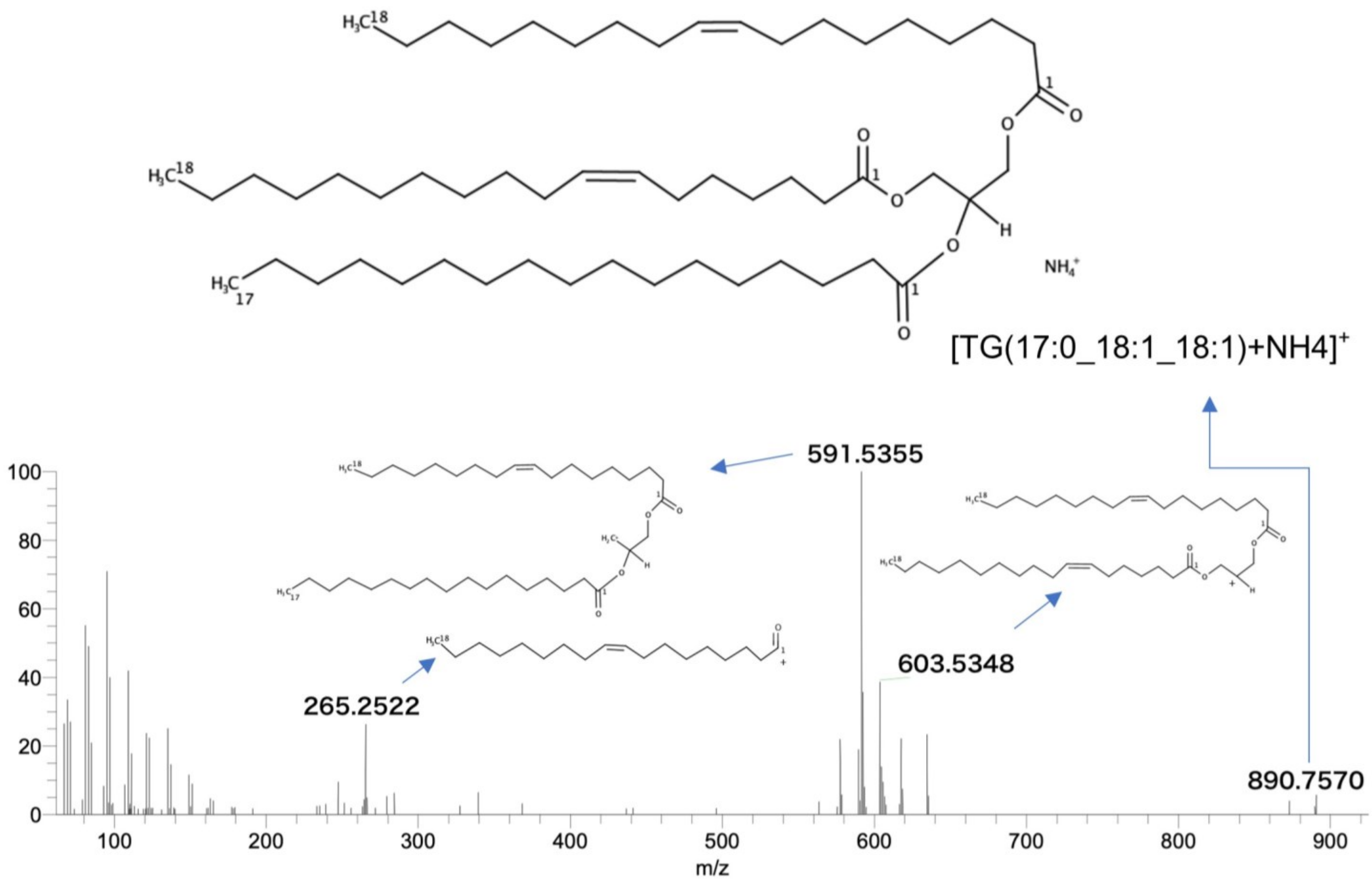

Supplemental Figure 7. MS/MS analysis for *m/z* 890.8180 (ID: 2279). The observed fragment ions were compatible with fatty acid side chains from [TG(17:1_18:1_18:1)+NH4]⁺.
Abbreviations: ID, identical number; MS/MS, tandem mass spectrometry; *m/z*, mass to charge ratio; TG, triglyceride.

Supplemental Table 1

	Lipid markers	TTF-1		p40	
		rS	*P*-value	rS	*P*-value
Positive markers for ADC	$[SM(d20:3\_22:0)+H]^+$	0.523	< 0.001*	-0.468	0.001*
	$[CL(18:2\_18:2\_18:2\_18:2)-H]^-$	0.332	0.028*	-0.542	< 0.001*
	$[SM(d40:1)+HCOO]^-$	0.374	0.012*	-0.376	0.012*
Positive markers for SQCC	$[AEA(18:1)+NH4]^+$	-0.558	< 0.001*	0.670	< 0.001*
	$[AEA(20:1)+NH4]^+$	-0.442	0.003*	0.604	< 0.001*
	$[TG(18:1\_17:1\_18:1)+NH4]^+$	-0.425	0.004*	0.515	< 0.001*
	$[TG(17:0\_18:1\_18:1)+NH4]^+$	-0.342	0.023*	0.500	< 0.001*

**P*-values < 0.05

Abbreviations: ADC, adenocarcinoma; AEA, anandamide; CL, cardiolipin; rS, Spearman's rank correlation coefficient; SM, sphingomyelin; SQCC, squamous cell carcinoma; TG, triglyceride; TTF-1, thyroid transcription factor-1.

Supplemental Table 2

Characteristics	ADC (n=6)	SQCC (n=9)
Degree of differentiation		
well	2 (33.3%)	1 (11.1%)
moderate	2 (33.3%)	5 (55.6%)
poor	2 (33.3%)	3 (33.3%)
Histologic subtype of ADC		
Lepidic	1 (16.7%)	-
Papillary	2 (33.3%)	-
Acinar	1 (16.7%)	-
Solid	2 (33.3%)	-
Immunohistochemistry		
TTF-1		
Diffuse positive	0	0
Focal positive	3 (50.0%)	3 (33.3%)
Negative	3 (50.0%)	6 (66.7%)
p40		
Diffuse positive	0	1 (11.1%)
Focal positive	0	7 (77.8%)
Negative	6 (100%)	1 (11.1%)

Abbreviations: ADC, adenocarcinoma; SQCC, squamous cell carcinoma; TTF-1, thyroid transcription factor-1.

Supplemental Table 3

Characteristics	Difficult (n=5)	Feasible (n=4)	P-value
Median specimen weight (mg) (range)	15.2 (1.5-69.6)	17.4 (13.5-24.4)	0.670
Median age (range)	70 (65-83)	75 (54-81)	0.994
Median Brinkman index (range)	1530 (920-3000)	1277 (700-1770)	0.280
Pathological stage			0.381
ⅠA	2 (40.0%)	1 (25.0%)	
ⅠB	1 (20.0%)	3 (75.0%)	
ⅡA	0	0	
ⅡB	2 (40.0%)	0	
ⅢA	0	0	
Degree of differentiation			0.167
well	0	0	
moderate	2 (40.0%)	3 (75.0%)	
poor	3 (60.0%)	0	
Immunohistochemistry			
TTF-1			1.000
Diffuse positive	0	1 (25.0%)	
Focal positive	2 (40.0%)	4 (100%)	
Negative	3 (60.0%)	0	
p40			1.000
Diffuse positive	0	0	
Focal positive	1 (20.0%)	3 (75.0%)	
Negative	3 (60.0%)	1 (25.0%)	

Abbreviation: TTF-1, thyroid transcription factor-1.

Supplemental Figure 8

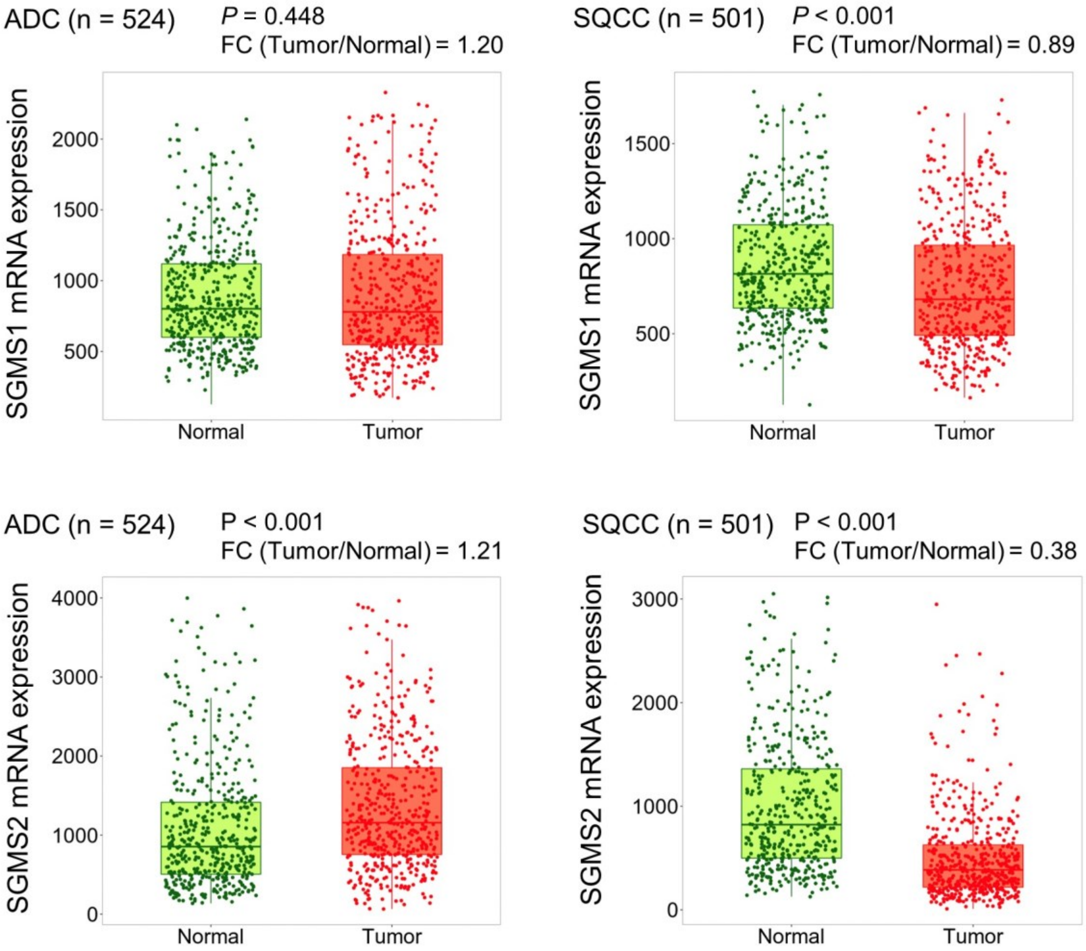

Supplemental Figure 8. mRNA expression level of SGMS1 and SGMS2. SGMS1 mRNA expression of tumor tissue was significantly lower than normal tissue in lung SQCC, while lung ADC showed no significant difference (upper panels). SGMS2 mRNA expression of tumor tissue was significantly lower than normal tissue in lung SQCC, while that was significantly higher than normal tissue in lung ADC (lower panels). Abbreviations: ADC, adenocarcinoma; FC, fold change; SGMS1, sphingomyelin synthase 1; SGMS2, sphingomyelin synthase 2; SQCC, squamous cell carcinoma.

Supplemental Figure 9

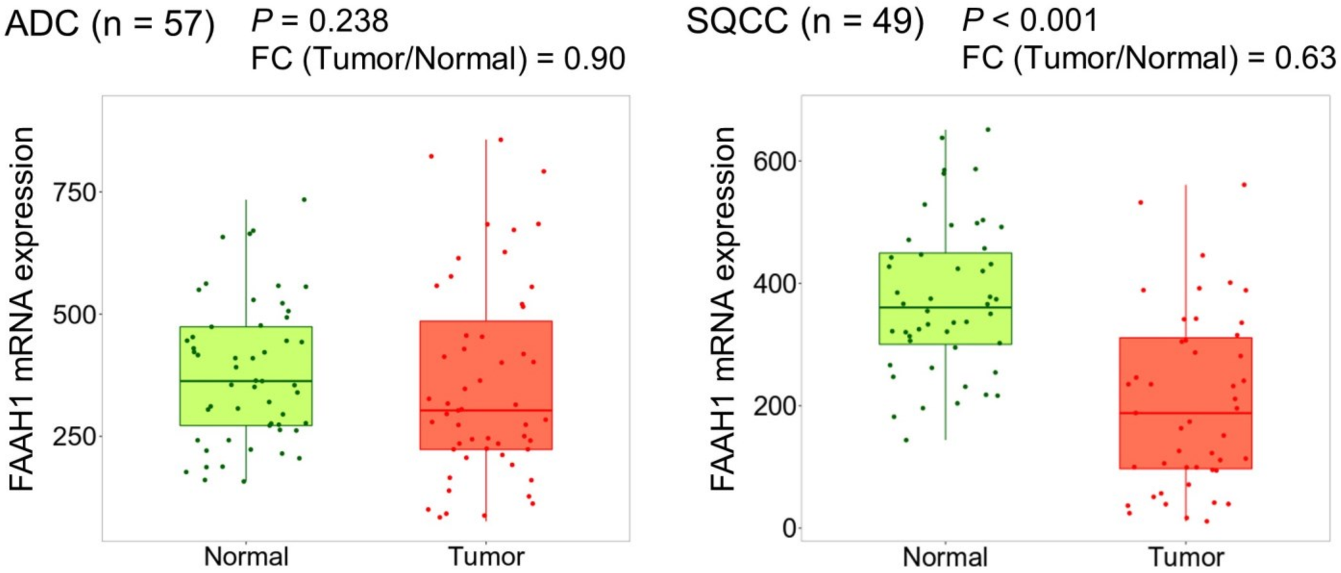

Supplemental Figure 9. The mRNA expression level of FAAH1. FAAH1 mRNA expression of tumor tissue was significantly lower than normal tissue in lung SQCC, while lung ADC showed no significant difference. Abbreviations: ADC, adenocarcinoma; FAAH1, fatty acid amide hydrolase 1; FC, fold change; SQCC, squamous cell carcinoma.

Supplemental Figure 10

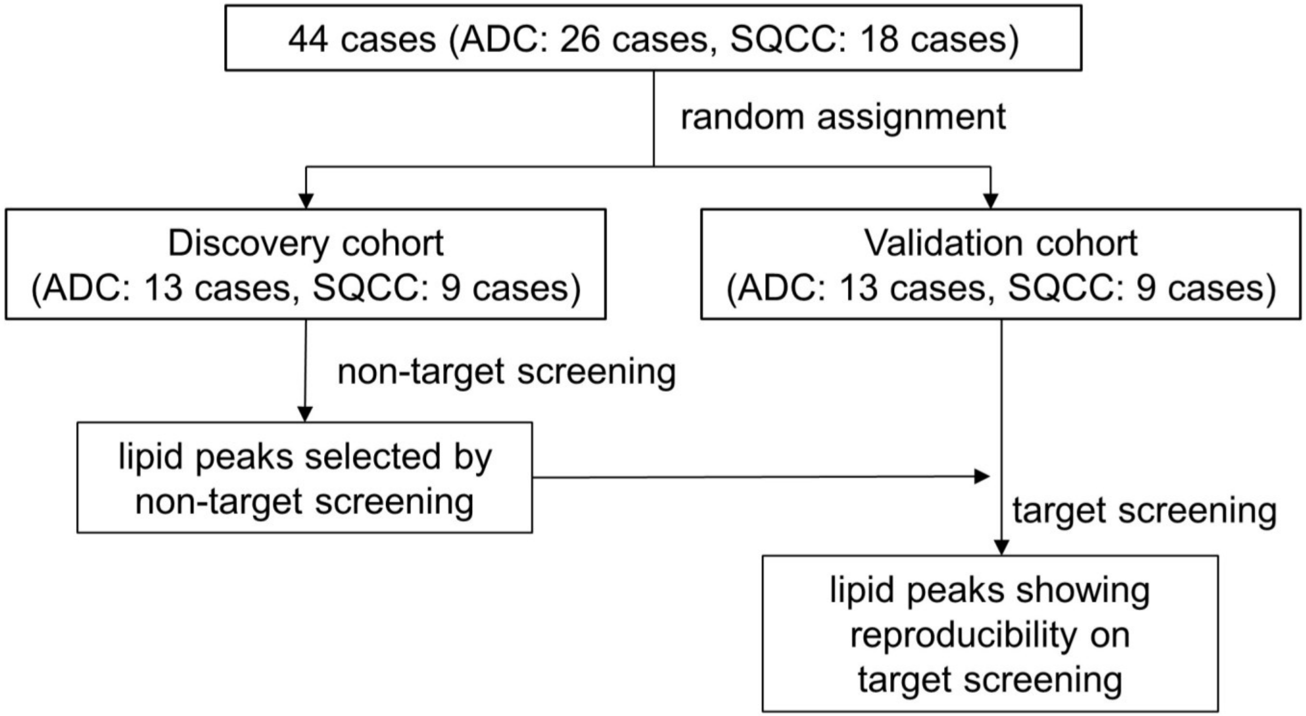

Supplemental Figure 10. A total of 44 lung cancer tissue samples (26 ADC and 18 SQCC cases) obtained from lung resection between January 2013 and December 2016 at Hamamatsu University Hospital were enrolled. The samples were randomly divided into discovery and validation cohorts. In the discovery cohort, lipid peaks with significantly different levels between ADC and SQCC cases were selected by a non-target screening. Subsequently, target screening focusing on the selected lipid peaks was performed on the validation cohort to examine their reproducibility.
Abbreviations: ADC, adenocarcinoma; SQCC, squamous cell carcinoma.
